# The use of the DTO™ hybrid dynamic device: a clinical outcome- and radiological-based prospective clinical trial

**DOI:** 10.1186/s12891-018-2103-x

**Published:** 2018-06-21

**Authors:** Christian Herren, Rolf Sobottke, Miguel Pishnamaz, Max Joseph Scheyerer, Jan Bredow, Leonard Westermann, Eva Maria Berger, Stavros Oikonomidis, Peer Eysel, Jan Siewe

**Affiliations:** 1 Department for Trauma and Reconstructive Surgery, University Hospital RWTH Aachen, Pauwelsstraße 30, 52074 Aachen, Germany; 2 Department of Orthopaedic and Trauma Surgery, University of Cologne, Joseph-Stelzmann-Straße 9, 50924 Cologne, Germany; 3 Department of Orthopaedic Surgery, Rhein-Maas Klinikum GmbH, Mauerfeldchen 25, 52146 Würselen, Germany; 4 Center for Spinal Surgery, Schön Klinik Düsseldorf, Schön Klinik Düsseldorf SE & Co. KG, Am Heerdter Krankenhaus 2, 40549 Düsseldorf, Germany

## Abstract

**Background:**

The purpose of this study was to assess the radiological and clinical outcome parameters following lumbar hybrid dynamic instrumentation with the focus on the adjacent segment degeneration (ASD) and adjacent segment disease (ASDi).

**Methods:**

In this prospective trial all patients presenting with degenerative changes to the lumbar spine have been included. Precondition was a stable adjacent level with/without degenerative alteration. The elected patients underwent a standardised fusion procedure with hybrid instrumentation (DTO™, Zimmer Spine Inc., Denver, USA). Patients’ demographics have been documented and the follow-up visits were conducted after 6 weeks, and then stepwise after 6 up to 48 months. Each follow-up visit included assessment of quality of life and pain using specific questionnaires (COMI, SF-36, ODI) and the radiological evaluation with focus on the adjacent level alterations.

**Results:**

At a mean follow up of 24 months an incidence of ASD with 10.91% and for ASDi with 18.18% has been observed. In 9% a conversion to standardised fusion was needed. There was a high rate of mechanical complication: (1) screw loosening (52.73%), (2) pedicle screw breakage (10.91%), and (3) rod breakage (3.64%) after a follow up of a maximum of 60 months. There were no significant difference of COMI, ODI and SF-36(v2) in comparison to all groups but all 55 patients showed a clinical improvement over the time.

**Conclusion:**

The dynamic hybrid DTO™ device is comparable to the long-term results after standardised fusion procedure, while a high rate of mechanical complication decreased the initial benefit.

**Trial registration:**

This trial was registered at the ClinicalTrials Register (#NCT03404232, 2018/01/18, registered retrospectively).

## Background

The most common age-related change to the spinal column is degenerative erosion that might lead to lumbar spinal stenosis (LSS). LSS might be accompanied by instability is commonly treated by open posterior decompression and additional fusion procedure [1–3]. Using a pedicle screw/rod-system in combination with an intervertebral cage (TLIF-, PLIF-technique) is a well-established procedure but a still controversial one due to a high revision rate up to 36% in certain circumstances [4]. The development of the adjacent segment degeneration (ASD), i.e. the known later complication in the terms of radiological detectable segment alteration, might outweigh the initial good clinical benefit to the patients following the fusion procedure [5–7]. Adjacent segment disease (ASDi) results in new clinical symptoms that are detectable adjacent to the previously fused segment. Alteration of biomechanics at the superior mobile segment is thought to accelerate degeneration in adjacent motion levels. Ha et al. described increased segmental mobility and changed contact patterns within the adjacent facet joint [8]. Hayes et al. found increased motion in adjacent level when L3-L4 was fused and this motion correlated with lower back pain [9]. Other studies found increased intradiscal pressure and shear loading within adjacent segments in cadaveric and finite element models [10–12]. Radiological signs of ASD may be more likely to occur when the adjacent segment has already signs of previous degeneration before undergoing fusion procedure [6, 13]. In recent years dynamic hybrid devices were developed to decrease the hypermobility of the adjacent level after fusion prophylactically. The aim is to imitate the physiological behaviour of the spine and to reduce load shearing on the adjacent segment that could hinder or retard the progression of adjacent segment degeneration [14]. One of the range-of-motion (ROM)-preserving dynamic instrumentation devices is the Dynesys-Transition-Optima (DTO™, Zimmer Spine Inc., Denver, USA). It combines rigid stabilisation with dynamic instrumentation of the segment superior to the rigid instrumented level. The patients with critical spinal instability and beginning adjacent segment alteration benefits from the rigid fusion and the additional performed decompression within the dynamic instrumented level. This might lead to a reduction of leg and back pain while preserving the ROM within the adjacent segment. However, there is no convincing evidence that dynamic hybrid devices provide any clinical benefit to the patient [7].

The purpose of the present prospective clinical trial was to determine the effectiveness and safety of the posterior dynamic instrumentation procedure adjacent to the rigid lumbar fusion in patients who were affected with degenerative lumbar alteration. This study focussed on the patient-related clinical outcomes measurements (COMI, ODI, SF-36) and the radiological detectable changes through a maximum follow-up of 60 months. We assume that the dynamic hybrid instrumentation procedure reduces the risk of (1) radiological detectable adjacent segment degeneration and (2) clinical assessable adjacent segment disease in a long-term follow up.

## Methods

A prospective non-randomised single-center study was performed to evaluate a cohort of patients who underwent posterior dynamic instrumentation adjacent to rigid lumbar fusion procedure. All patients with LSS, previous lumbar fusion procedure, degenerative lumbar disc disease, and/or spondylolisthesis were included. Precondition in all cases was a stable adjacent segment with/without degenerative alteration. Demographic data, comorbidities, preoperative symptoms and the instrumented lumbar segment were documented.

### Groups

Due to the heterogeneity of the patient’s cohort the included patients were divided into 3 groups: (1) patients with their first surgical intervention at the lumbar spine, (2) patients with a previous surgical decompression but non-fusion procedure after LSS and (3) patients with medical history of PLIF-/TLIF-technique and later onset of symptomatic ASD within the superior adjacent segment.

### Implant

The Dynesys DTO™ device (Zimmer Spine, Inc.) is a pedicle screw/rod based dynamic stabilisation system that combines the well-known Dynesys dynamic stabilisation system (DDSS) and the OPTIMA ZS spinal system. It is made of a combined 100 mm Polyehtylenterephthalate (PET) Dynesys cord and a titanium rod (Ø 6.0 mm). The DDSS is a device for the whole lumbar spine (L1-S1) while the Dynesys is placed cranially to the OPTIMA ZS system. The rigid part is instrumented between vertebra L2 and S1. The operative procedure includes a standard posterior instrumentation through a traditional midline approach.

### Follow-up visit

Follow-up visit is planned after 6 weeks and after 6, 12, 24, 36 and 48 months. During follow-up visit three different patient-dependent objective outcome measurement instruments were used: Core Outcome Measures Index (COMI), Oswestry low back pain disability index 2.1 (ODI) and Short-Form (36) Health Survey Version 2 (SF-36v2). Euro-QUOL (EQ-5D) superseded the SF-36 on 2013. For all patients included before 2013 the SF-36 questionnaire was used and after 2013 all patients received the new EQ-5D questionnaire.

The *COMI* is a short, self-administered outcome instrument consisting of seven questions to evaluate the five dimensions pain, back-related function, symptom-specific well-being, general quality of life and -disability (social and work). Two pain graphic rating scales (GRS 0–10 points) capture back and leg pain, and all other items use a 5-point adjectival scale. Obtaining the summary score the average of the scores for all five dimensions (each transformed to 0–10) is calculated.

The *SF-36v2* questionnaire is a survey of patient health and an independent measurement instrument that estimates the patient-reported subjective quality of life. It consists of 36 items subdivided in eight scaled scores. Each scale is transformed into a 0–100 scale. The lower the score the lower the disability. In contrast, the *ODI* and *EQ-5D* are disease-dependent measurement tools.

### Radiological assessment of the adjacent segment

In addition to the evaluation questionnaires, radiological imaging of the lumbar spine was performed (standard radiographs of the lumbar spine in a.p.- and lateral view). Assessment of the implant (implant failure: yes/no? and signs for implant loosening: yes/no?) and assessment of the adjacent segment (degenerative alteration: yes/no?) was conducted particularly. Radiological instability of the adjacent level was predefined as: (1) spondylolisthesis > 4 mm, (2) segmental kyphosis > 10°, (3) hypermobility, (4) collapsed disc space, (5) coronar translation > 3 mm, (6) disc wedging > 5° and/or degradation of > 2 scoring grades in the Weiner classification (Table 1). The Weiner classification is subdivided into 4 grades [15].

**Table 1:** Summary of the Weiner classification system

	Degeneration	Disc height	Spur formation	Listhesis
Grade 0	none	normal	none	none
Grade 1	mild	< 25% narrowing	small	none
Grade 2	moderate	25–75% narrowing	moderate	3-5 mm
Grade 3	advanced/high	> 75% narrowing	large	> 5 mm

All radiological obtained degeneration alterations were compared to preoperative images.

### Data and statistical analysis

All data analyses were performed with SPSS statistical software (IBM SPSS, Inc., Chicago, USA). Preoperative data were compared to postoperative findings at 12,24,36 and 48 months. A one-way ANOVA was used for the comparison of the different patients’ groups. The paired t-test was used for the analysis of the different outcome parameters in each group. The Logrank test was used to compare the treatment groups in terms of survival time to instability measured by the Kaplan-Meier method. A probability value (*p*) < 0,05/4 = < 0,0125 was considered statistically significant after the Bonferroni correction.

### Ethical statement

The study complies the principles of the Declaration of Helsinki (2013) and was approved by the local ethical committee (#09812, University Hospital Cologne). This trial was registered at the ClinicalTrials Register (#NCT03404232, 2018/01/18, registered retrospectively). Written informed consent was available for each participant who was involved in this study. Each participant gave his consent for the use of the anonymised data including demographic data, medical images and clinical scores.

## Results

A total of 55 patients met the inclusion criteria and were included in the study. A summary of the patients’ demographics and group details is shown in Table 2.

**Table 2:** Patients’ demographics and group details

Characteristics	Group 1	Group 2	Group 3
Patients (*n* = 55)	14	18	23
Female (*n* = 36)	10	12	14
Male (*n* = 19)	4	6	9
Mean age in years (range)	65.50 (47–84)	71.22 (60–80)	70.09 (55–81)
Follow up (mean in months)	26	32.80	35.42
Dynamic stabilised segments			
L4/5	4	3	0
L3/4	6	7	5
L2/3	3	7	12
L1/2	1	1	6

### Outcomes and quality of life

The mean follow-up time for the assessment in this study population was 31.40 (range 1.5–60) months. After 4 years the lost to follow-up amounted to 45.45% (*n* = 25/55) and was determined as being acceptable for further statistical analysis. At the time of 60 months a very high lost to follow-up rate was detectable (63.64%) not being considered for the further analysis. The lost to follow-up rate was balanced within the three groups.

**Fig. 1:**
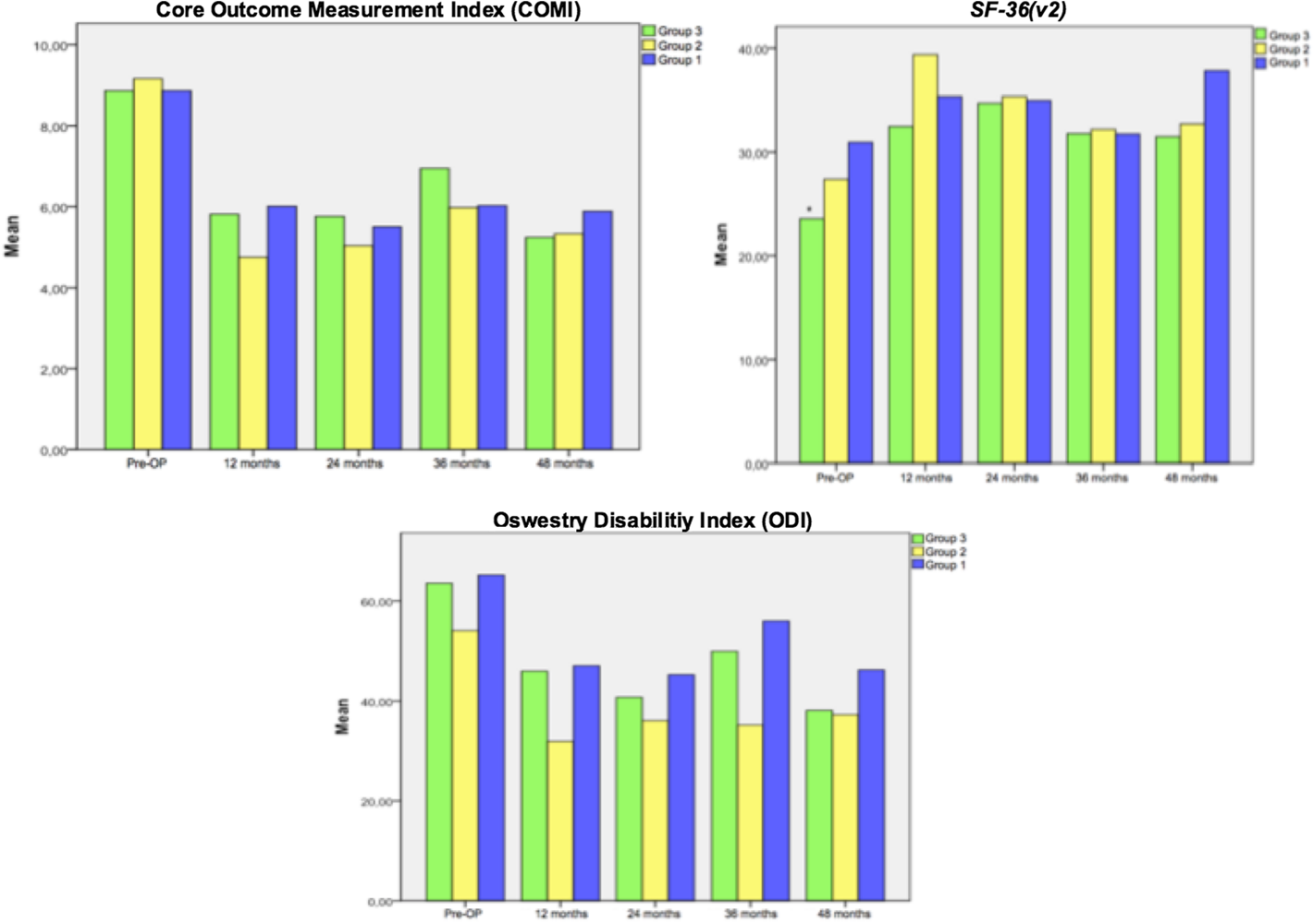
Analysis of the different questionnaires. Significant parameters were marked with ‘*’

Between the three groups, there were no significant changes detectable regarding the mean scores of the COMI, *SF-36(v2)* and ODI over the entire follow-up time (Table 3). Looking at the total scores of the above-mentioned assessment parameters all patients benefited from the surgical treatment (Fig. 1).

**Table 3:** Analysis of the different assessment parameters (COMI, ODI, *SF-36(v2)*) between the three groups from the preoperative condition up to the 48-months follow-up

Outcome	Group 1	Group 2	Group 3	*p*-value
COMI				
preoperative	8.86 ± 0.625	9.16 ± 0.992	8.86 ± 1.294	0.639
12 months	6.01 ± 2.510	4.75 ± 3.178	5.81 ± 2.796	0.504
24 months	5.50 ± 3.838	5.04 ± 2.334	5.76 ± 2.881	0.84
36 months	6.03 ± 2.793	5.98 ± 3.460	6.95 ± 2.695	0.80
48 months	5.89 ± 3.359	5.33 ± 2.344	5.24 ± 2.946	0.934
ODI				
preoperative	65.21 ± 15.793	54.06 ± 12.497	63.57 ± 15.791	0.074
12 months	47.11 ± 19.701	31.92 ± 18.936	46.00 ± 18.796	0.093
24 months	45.29 ± 26.625	36.10 ± 14.985	40.75 ± 21.246	0.67
36 months	56.00 ± 30.199	35.20 ± 25.519	50.00 ± 15.895	0.343
48 months	46.25 ± 26.862	37.29 ± 16.958	38.13 ± 19.982	0.761
*SF-36(v2)*				
preoperative	30.95 ± 5.657	27.37 ± 6.512	23.56 ± 6.137	0.022^*^
12 months	35.36 ± 15.514	39.38 ± 10.482	32.47 ± 9.778	0.290
24 months	34.97 ± 14.243	35.36 ± 11.051	34.71 ± 12.032	0.991
36 months	31.75 ± 15.627	32.20 ± 16.085	31.78 ± 8.411	0.998
48 months	37.83 ± 16.574	32.73 ± 5.192	31.50 ± 8.786	0.587

Significant differences are marked with ‘*’

**Fig. 2:**
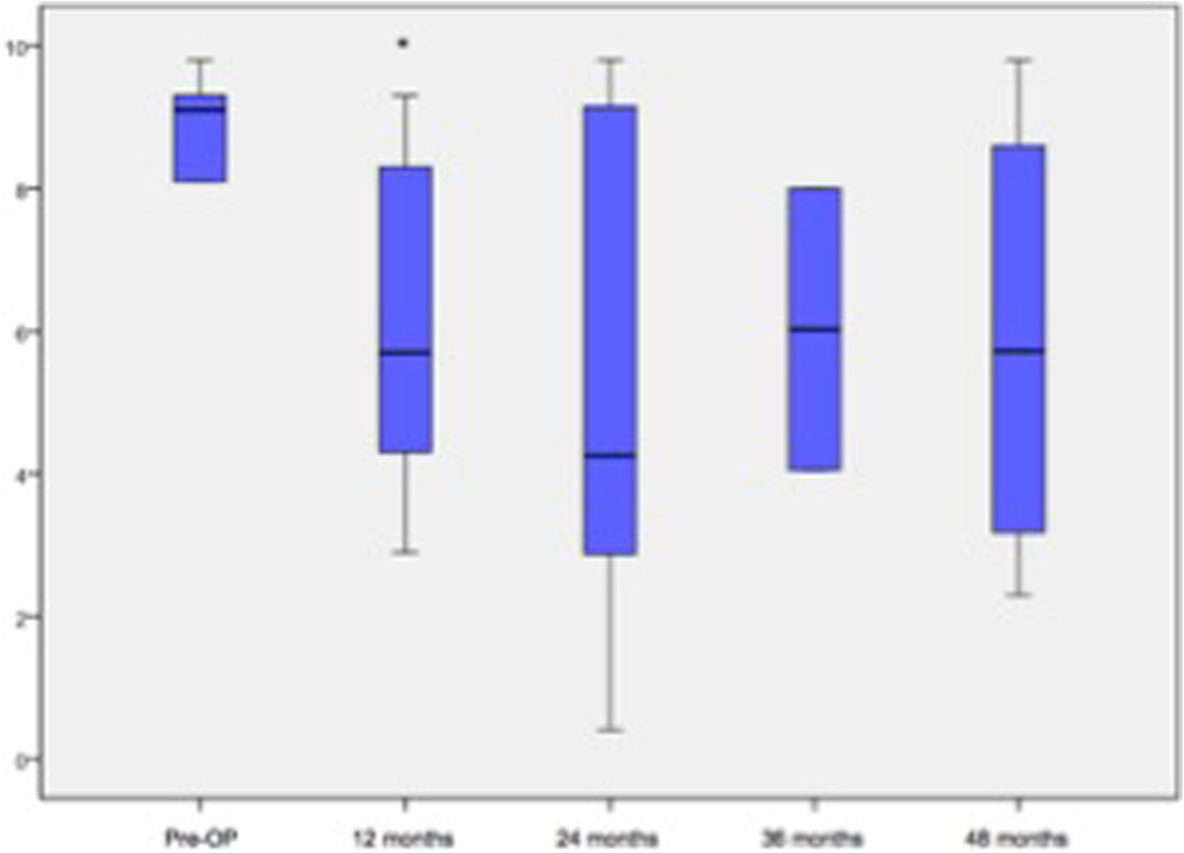
Analysis of the COMI within group 1. Significant parameters are marked with ‘*’

Within group 1, the COMI improved significantly from a preoperative mean of 8.9 ± 0.625 to a mean of 6.0 ± 2.510 after 12 months (*p* = 0.009; Fig. 2). After 12 months, the mean ODI values tended to be significant in comparison to the preoperative assessment (47.1 ± 19.701 vs. 65.2 ± 15.793; *p* = 0.013). The values from the *SF-36 (v2)* questionnaire improved non-significantly from a preoperative mean of 30.9 ± 5.657 to a mean of 37.8 ± 16.574 points at the last available follow up (48 months, *p* = 0.179).

**Fig. 3:**
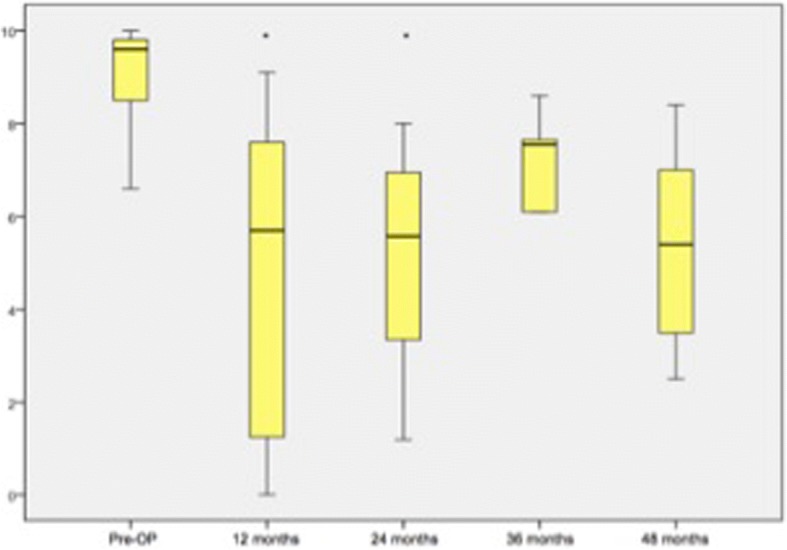
Analysis of the COMI (group 2) over the time. Significant parameters are marked with ‘*’

**Fig. 4:**
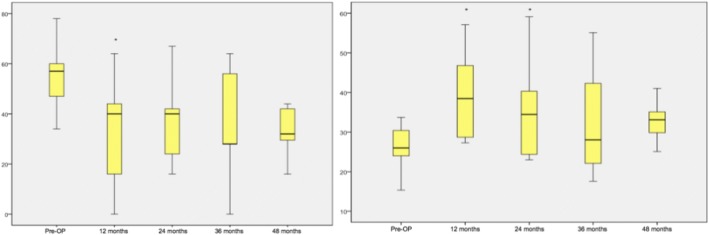
Analysis of group 2 of the ODI (left) and the *SF-36v2* (right). Significant parameters are marked with ‘*’

Within group 2 the analysis of the mean COMI scores showed that the patients tended to be satisfied in comparison to the preoperative condition at the last available follow up with 48 months (9.2 ± 0.992 vs. 5.3 ± 2.344, *p* = 0.018). At 12 months and 24 months the COMI improved to a mean of 4.8 ± 3.178 and 5.0 ± 2.334 significantly in comparison to the preoperative baseline (*p* < 0.001, Fig. 3). The analysis of the ODI and *SF-36(v2)* scores showed a significant progress regarding the activities of daily living at 1 year (ODI: 31.9 ± 18.936, *p* = 0.001; *SF-36(v2)*: 39.3 ± 10.482, *p* = 0.001) and as well for the *SF-36(v2)* after 24 months (35.4 ± 11.051, *p* = 0.008; Fig. 4).

**Fig. 5:**
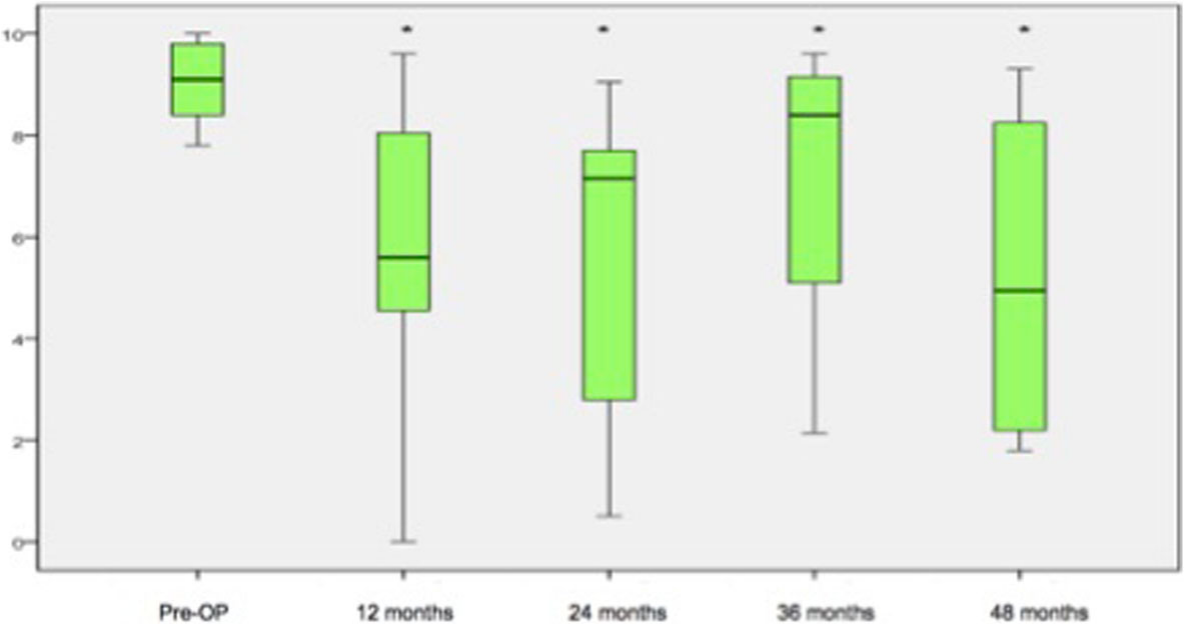
Analysis of the COMI questionnaire (group 3). Significant parameters are marked with ‘*’

**Fig. 6:**
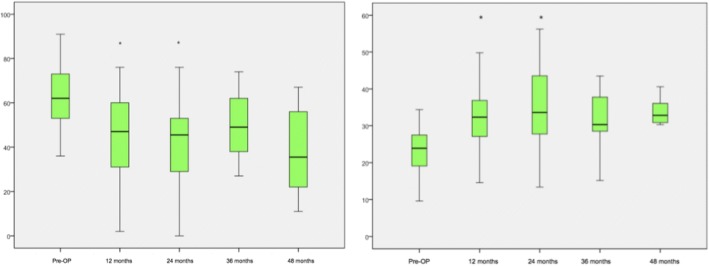
Analysis of group 3 of the ODI (left) and the *SF-36v2* (right). Significant parameters are marked with ‘*’

Within group 3 the analysis of the COMI values showed a significant improvement over the time (Fig. 5). The mean COMI values improved from 8.9 ± 1.294 to 5.8 ± 2.796 after 12 months (*p* < 0.001). After 24, 36 and 48 months the COMI values developed significantly from 5.8 ± 2881 (*p* = 0.001) and 6.9 ± 2.695 (*p* = 0.010) to 5.2 ± 2.946 (*p* = 0.011). Both, the analysis of the ODI and *SF-36(v2)* results showed a significant improvement in comparison to the preoperative condition especially within the first 2 years (Fig. 6). Within the first 24 months, the ODI values changed stepwise from 63.6 ± 15.791 to 46.0 ± 18.796 (*p* = 0.002) and 40.7 ± 21.246 (*p* = 0.003). At the last available follow-up the assessment of the ODI showed that the patients tended to be satisfied (48 months: 38.1 ± 19.982, *p* = 0.027). The analysis of the *SF-36(v2)* values showed a significant improvement after 12 (32.5 ± 9.778, *p* = 0.004) and 24 months (34.7 ± 12.032, p = 0.003) in comparison to the preoperative condition (23.6 ± 6.137).

### Radiological outcomes

The overall rate of ASDi in all groups was 18.18% within the segment superior to the dynamic instrumented level (sSDL). Inside of a two years follow up, 70% of the patients developed clinical signs of ASDi. Looking at the global differences, 21.4% (*n* = 3) of all patients treated first time at the lumbar spine (group 1), 18.75% (*n* = 3) of patients with a previous surgical decompression but non-fusion procedure after LSS (group 2) and 17.4% (*n* = 4) of patients with medical history of PLIF-/TLIF-technique (group 3) developed a clinical symptomatic ASDi. Due to ASDi, 9% of the patients needed a revision surgery in conventional PLIF-technique of the involved segment.

**Fig. 7:**
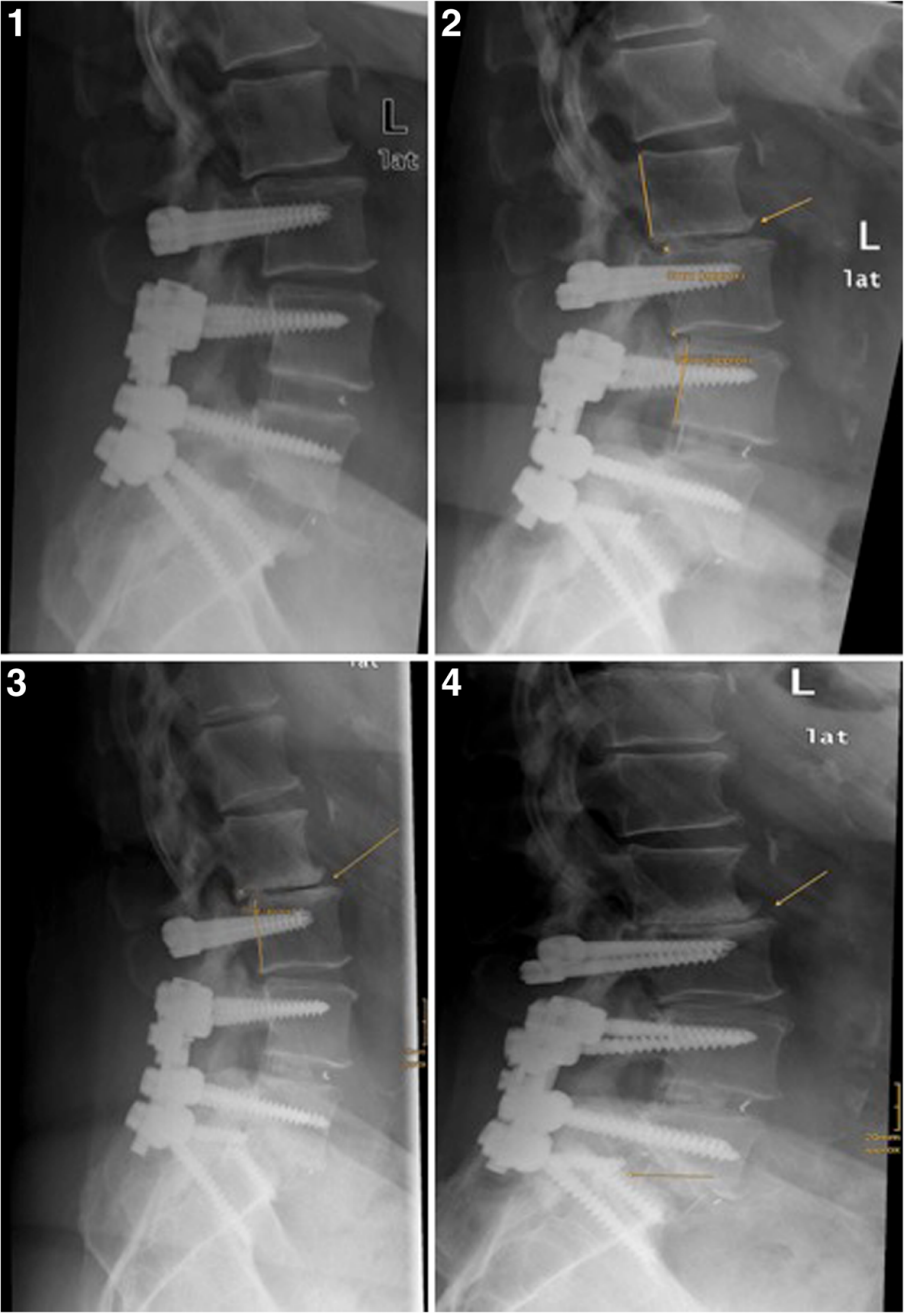
Follow-up of a female patient and onset of radiological detectable ASD without ASDi in 3 steps during 60 months. (1) postoperative plain radiographs of the lumbar spine after PLIF L5/S1 and L4/5 with dynamic instrumentation of L3/4. (2) 12 months follow up: early signs of listhesis, disc space narrowing and beginning spurs formation (L3/4, yellow arrow). (3) 36 months follow up: progressing listhesis and osteochondrosis within the segment superior to the dynamic instrumented segment (yellow arrow). (4) 48 months follow up: listhesis and advanced degeneration of L3/4 (yellow arrow)

Radiological detectable ASD was found in 10.91% (*n* = 6). In contrast to the ASDi, ASD was detectable in both segments – within the sSDL and within the dynamic instrumented level (DL). In total, a listhesis > 4 mm has been observed in 5 cases (3 sSDL vs. 2 DL). One of these patients showed a degradation of > 2 scoring grades in the Weiner classification and the presence of gas within the disc space of the sSDL (Fig. 7). Hypermobility with coronar translation was found in another patient and in one patient a disc wedging > 5° has been observed within the sSDL due to the listhesis.

Comparing with the three different groups, none of these results were significant (Log-Rank-Test: ASDi *p* = 0.799; ASD *p* = 0.508).

### Implant loosening

**Fig. 8:**
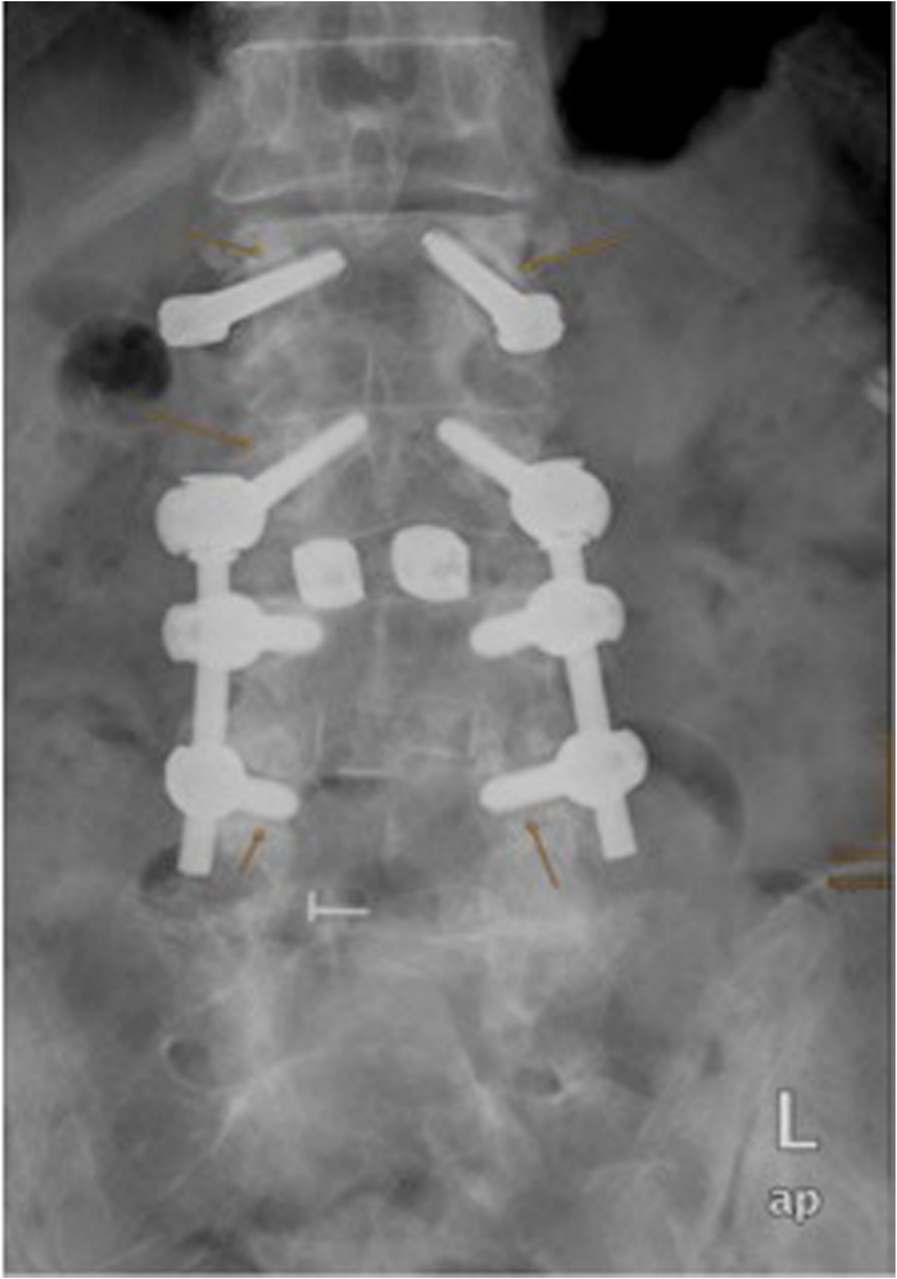
Radiograph of the lumbar spine in ap-view. Slight signs of implantat loosening within L2,L3 and L5 (marked with a yellow arrow)

Analysing the plain radiographs showed an overall rate of pedicle screw loosening in 52.73% (*n* = 29) of the patients. Pedicle screw loosening in multiple levels was present in 6 patients (Fig. 8). A radiological detectable ASD (*n* = 5) and a clinical ASDi (*n* = 5) has been observed in 34.48% (*n* = 10) of these patients.

### Implant failure

**Fig. 9:**
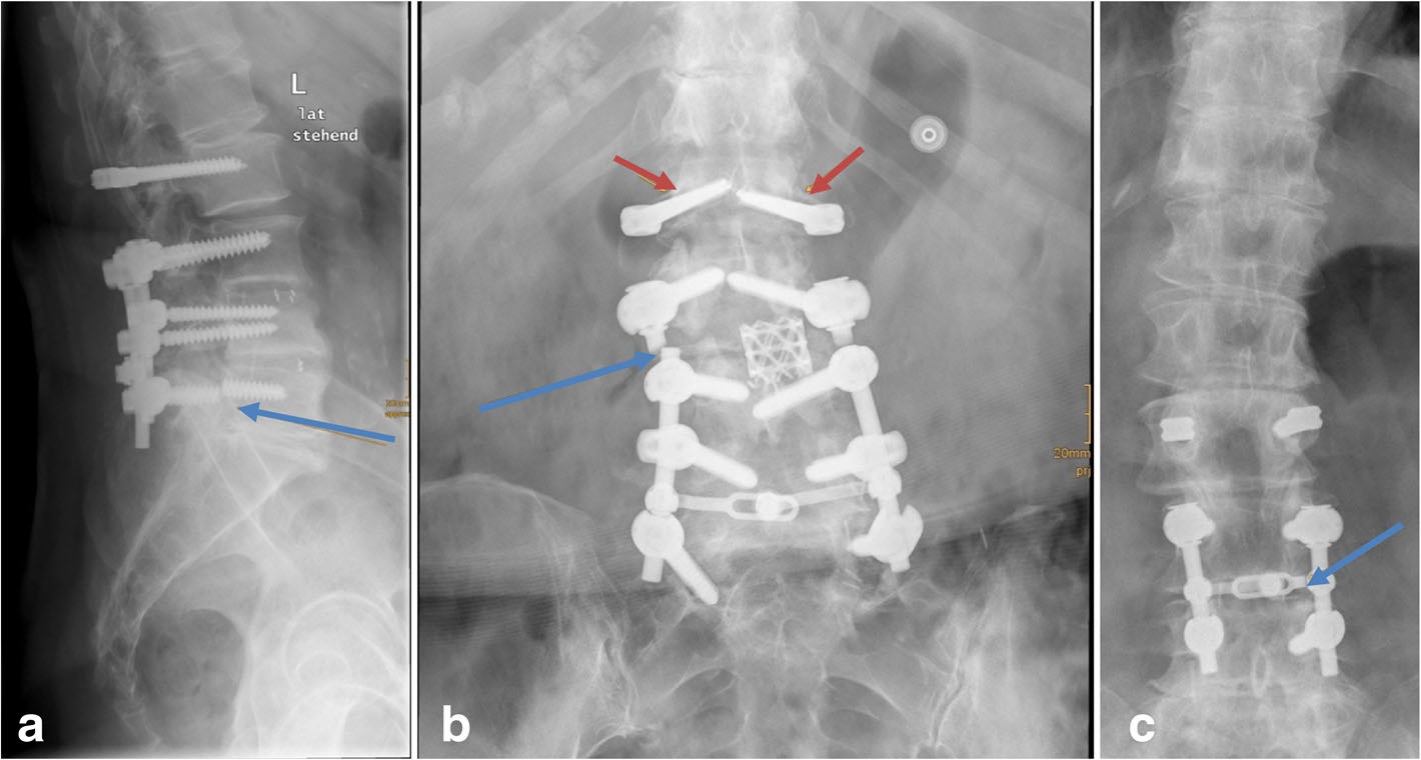
Radiographs representing the three types of observed implant failure: (**a**) breakage of the left inferior pedicle screw (blue arrow); (**b**) breakage of the titanium rod inferior to the dynamic instrumented level (blue arrow) and loosening of the Dynesys screws (red arrows); (**c**) breakage of the crossbar (blue arrow)

In total, 8 cases (14.55%) of implant failure of the Dynesis device has been observed with a homogeneous contribution within the three groups (Fig. 9):

- Group 1: breakage of 2 pedicle screws (rigid part of the Dynesys system)
- Group 2: breakage of 3 pedicle screws (rigid part of the Dynesys system) and breakage of the horizontal linking rod within the rigid instrumented level
- Group 3: breakage of 1 pedicle screw (rigid part of the Dynesys system) and breakage of the titanium longitudinal rod within the rigid instrumented part.

All six cases of pedicle screw breakage were present within the inferior rigid instrumented vertebra.

### Vertebral fracture

**Fig. 10:**
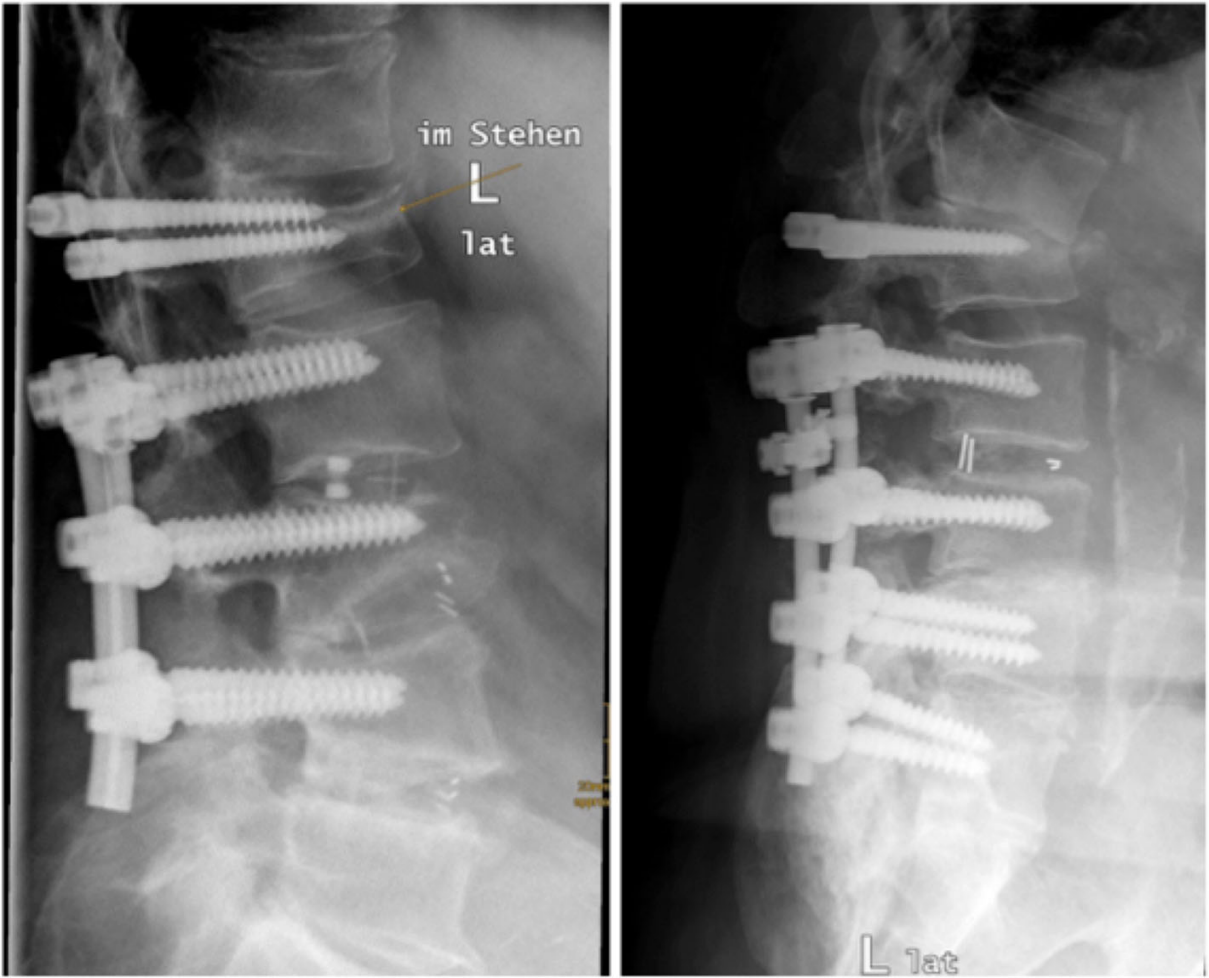
Radiographs in lateral view showed a vertebral fracture of the topping off vertebra (L1)

During follow up, in 10.91% (*n* = 6) of all surgical treated patients a vertebral fracture occurred. All fractures were present at L1 and in four cases the dynamic fixed vertebra was affected. Looking at the three groups, 8 patients were dynamic instrumented within the segment L1/2 and in 50% a vertebral fracture of the dynamic instrumented vertebra occurred (Fig. 10).

## Discussion

Lumbar spinal fusion is the golden standard for treatment of spinal instability [16]. Fusion procedure might lead to immediate good clinical results and moderate long-term benefits due to complications and development of ASD and ASDi [4]. Clinical rates of ASD has been reported up to 31.3% and it could be shown that patients with ASD had a significantly worse ODI score than patients without ASD after posterior fusion [17]. The development of dynamic hybrid instrumentation has been thought to reduce the risk of ASD and ASDi. However, there is no convincing evidence regarding the clinical and radiological benefits of the hybrid devices. Published research about pedicle screw-based hybrid dynamic instrumentation devices is rare and long-term data are even sparer.

In this study with a mean 31-months follow-up applying dynamic hybrid instrumentation surgery, all 55 patients treated by DTO™ hybrid instrumentation showed clinical improvement in COMI, ODI and *SF-36(v2)* over the time. Especially the patients with previous fusion procedure and later onset of symptomatic ASD within the superior adjacent segment benefited from the hybrid dynamic instrumentation. Similar results have been reported by Maserati et al.. They found a clinical improvement of the VAS of 24 patients underwent fusion and dynamic instrumentation with the DTO™ device during a mean follow up of 8 months [18]. In terms of the positive development of ODI, Baioni et al. supported the in this study presented results. In a cohort of 30 patients they showed an improvement of the mean ODI from 67.6 to 27.7 at the last available follow-up of 5 years (*p* < 0.05) [19]. In the literature, the pedicle-based dynamic instrumentation is reported as being equivalent to standardised fusion procedure. Putzier et al. showed no significant difference in long-term outcome (ODI and VAS) between standardised fusion and hybrid dynamic instrumentation after a maximum follow-up of 76.4 months [20]. One reason might be the additional decompression within the dynamic instrumented segment [21]. These results have also been confirmed by Schwarzenbach et al.. They reported in a population of 31 patients a significant improvement in postoperative ODI and VAS after instrumentation with Dynesys [22]. Hudson et al. found an improvement in ODI, VAS and *SF-36(v2)* during follow-up of 24 months after topping off dynamic instrumentation with Isobar TTL® (Scient’x, USA) [23].

In contrast to the above-mentioned studies the present results suggest that after hybrid dynamic instrumentation, the initial positive effect decreased during long-term follow-up up to 60 months. This might be caused by the development of increasing complication rates after dynamic hybrid instrumentation, especially the problem regarding screw loosing. We found a high incidence of screw loosening with 52.73%, observed in different levels of the instrumentation. This high rate might be caused by the higher age of the cohort (mean age 68.94 years) and it is well known that the osseous quality has a direct influence on the risk of screw loosening in the elderly [24]. Another reason is that the stiffness of the devices might be positively correlated with the rates of screw loosening. In contrast to this, a high implant stiffness might also be reasonable for the benefits after a short-term period in terms of the clinical outcomes as it preserves the preoperative distraction thus unloading the anatomic structures [21].

While revision rates of the well-known Dynesys device have been reported up to 34%, to date, there is minimal research regarding the DTO™ [25, 26]. Maserati et al. reported a non-implant dependant complication rate of 24% but they found no screw breakage or signs for implant loosening [18]. During a long-term follow up, another group found no signs of implant failure of the DTO™ within 5 years [19]. Contrary to this we found an overall rate of implant-associated complication up to 67.27% including cases of screw loosening. Screw breakages (10.91%) especially occurred within the fusion part of the instrumentation and two cases of breakage (3.64%) of the longitudinal rod and horizontal connecting rod have been observed after a follow up of a maximum of 60 months. Similar to our results, Hoff et al. found a high rate (30%) of implant breakage (rod and screws) by the use of the CD Horizon® Agile™ Spinal System (Medtronic, Memphis, TN, USA) topping-off device [27]. Putzier et al. presented a prospective clinical trial of the Allospine™ Dynesys® Transition System (Zimmer Co., Winterthur, Switzerland) which is described as the prototype of the DTO™ device. They found, both, screw breakages (*n* = 2, 9.09%) and one case of longitudinal rod breakage (4.55%) during a 6-years follow up [20]. It might be assumed that similar to the above mentioned topping-off devices the DTO™ leads to more biomechanical shear-load on the rigid instrumented segment due to shifting of the tensile and compression forces to the upper rigid transition screw of the construct.

The incidence of ASD in our population was 10.91 and 18.18% for ASDi. In comparison to standard lumbar fusion procedure the present overall rates were four (ASD) and three (ASDi) times lower [17]. The amount of patients converting to standard fusion was 9% over time and similar to the reported rates in other studies (6.67%) [19]. Kashkoush et al. reported a higher rate with 15% converting to traditional fusion procedure due to new symptoms or persistence of the preoperative complaints [25].

Similar to our findings, Hoff et al. observed a progression of the ASD superior to the dynamic instrumented level and assumed that the loading forces were shifted cranially due to the implant stiffness of the topping off device [27]. Interestingly, we observed a high rate of fractures adjacent to or within the dynamic instrumented vertebra (10.91%) that are not reported in the literature. One reason for this might be the poorer osseous quality combined with the implant stiffness shifting the shear-loads one level superior to the instrumented level. This problem is shown by several biomechanical in-vitro studies and from the biomechanical point of view it is not clear how far the hybrid devices reduce the force on the adjacent segment [28, 29].

### Limitations

There are limitations to our study. Due to the wide range regarding the study population age, a heterogeneous population has been analysed. Second, there is no control group in this study and due the fact that data were obtained from patients who underwent surgery in a single-center, there might be a selection bias. A further randomized controlled study in multiple hospitals should be conducted. Third, the influence of the sagittal balance and other pathoanatomic risk factors (facet tropism and sagittalisation, horizontalisation of the lamina) has not been observed. However, this is one of the first studies considering patient-oriented clinical outcomes and radiological effects regarding a follow-up up to 60 months.

## Conclusion

Despite the high rate of implant failures within the fusion and dynamic segment, surprisingly, the mechanical complication did not lead to a lower clinical outcome (COMI, ODI and *SF-36(v2)*) over the time. Furthermore, the development of ASD or ASDi has been observed superiorly to the dynamic section during long-term follow-up. Due to the implant stiffness the loading forces were simply shifted to the segment superior to the instrumented level. However, the present study cannot support the safety of dynamic hybrid devices in those cases if the reduction of ASD is the main target. Further biomechanical studies are needed for optimising the implant design to reduce the implant stiffness and improve the patients’ safety.

## Abbreviations

ASD: Adjacent segment degeneration
ASDi: Adjacent segment disease
COMI: Core outcome measures index
LSS: Lumbar spinal stenosis
ODI: Oswestry disability index
PLIF: Posterior lumbar interbody fusion
ROM: Range-of-motion
SF-36: Short-form 36
TLIF: Transforaminal lumbar interbody fusion
VAS: Visual analog scale
